# Evaluating the Implementation of Online Postal Self-Sampling for Sexually Transmitted Infections in England: Multisite Qualitative Study

**DOI:** 10.2196/72812

**Published:** 2025-09-09

**Authors:** Tommer Spence, Jo Gibbs, Geoff Wong, Alison Howarth, Andrew Copas, David Crundwell, Louise Jackson, Catherine H Mercer, Hamish Mohammed, Vanessa Apea, Sara Day, Jonathan Ross, Ann Sullivan, Andrew Winter, Claire Dewsnap, Fiona M Burns, Jessica Sheringham

**Affiliations:** 1 University College London London United Kingdom; 2 University of Birmingham Birmingham United Kingdom; 3 University of Oxford Oxford United Kingdom; 4 David Crundwell Corporate Affairs and Private Equity London United Kingdom; 5 UK Health Security Agency London United Kingdom; 6 Barts Health NHS Trust London United Kingdom; 7 Chelsea and Westminster Hospital NHS Foundation Trust London United Kingdom; 8 NHS Greater Glasgow and Clyde Glasgow United Kingdom; 9 Sheffield Teaching Hospitals NHS Foundation Trust Sheffield United Kingdom

**Keywords:** digital health, implementation, sustainability, sexually transmitted infections, sexual health services

## Abstract

**Background:**

Online postal self-sampling (OPSS) allows service users to screen for sexually transmitted infections (STIs) by ordering a self-sampling kit online, taking their own samples, returning them to a laboratory for testing, and receiving their results remotely. OPSS availability and use has increased in both the United Kingdom and globally the past decade but has been adopted in different regions of England at different times, with different models of delivery. It is not known why certain models were decided on or how implementation strategies have influenced outcomes, including the sustainability of OPSS in sexual health service delivery.

**Objective:**

This study aims to evaluate the implementation of OPSS in 3 case study areas of England, with a focus on the sustainability of implementation and the relationship between implementation strategies and outcomes.

**Methods:**

Qualitative data collection methods were used: interviews with staff and stakeholders involved in the implementation and delivery of OPSS, analysis of local implementation and national policy documents, and observations in sexual health clinics. Analysis of interviews and observations was undertaken using qualitative implementation science frameworks, including normalization process theory, the Consolidated Framework for Implementation Research, and the major system change framework. Documentary sources were used primarily to map processes over time and triangulate against interview and observational evidence.

**Results:**

Across the 3 case study areas, 60 staff and stakeholders were interviewed, 12 observations were conducted, and data from 86 documents were collated. Rather than being a discrete digital health intervention, we found that OPSS was part of—or occurred parallel to—major system changes in all areas. These changes were driven by budgetary pressures in all areas, but there was variation in other objectives used to rationalize the decision to adopt. The financial context and organizational relationships in each area determined the implementation strategies available to decision makers, how these strategies were enacted, and, in turn, led to different outcomes at different time points. OPSS implementation was not a one-off outcome but an ongoing process in response to changes in context, which in turn affected how staff perceived and engaged with OPSS. The COVID-19 pandemic had profound but divergent effects on OPSS implementation in each area, accelerating it in some contexts and reversing it in others.

**Conclusions:**

In this multisite case study, OPSS implementation was part of systems change to address a wider problem of insufficient funding to deliver sexual health care. Decisions about implementing OPSS were made before sufficient evidence was available to effectively guide the process. The resultant unintended consequences need acknowledgment to enable future commissioners and sexual health services to optimize sexual health service provision.

**International Registered Report Identifier (IRRID):**

RR2-10.1136/bmjopen-2022-067170

## Introduction

### Background

Online postal self-sampling (OPSS) is a digital health intervention that allows service users to screen for sexually transmitted infections (STIs) and blood-borne viruses (BBVs) by ordering a self-sampling kit online, taking their own samples, returning them to a laboratory for testing, and receiving their results remotely [1]. OPSS differs from self-testing in that samples are sent off for processing in a laboratory, rather than being processed within the test kit [1-3]. Results are usually communicated electronically, and users in need of treatment or partner notification are typically referred to clinic-based sexual health services [4].

Many settings globally are developing and implementing OPSS, with its availability and uptake having been accelerated during the COVID-19 pandemic [5-8]. The proportion of tests conducted via OPSS within England’s National Chlamydia Screening Programme, for example, rose from 19% in 2019 to 43% in 2023 [9]. This transition is occurring within the context of wider efforts to digitize health care; there is a national recommendation that OPSS should be provided as part of all sexual health services in England, while the World Health Organization recommends that self-sampling be provided alongside clinic-based sexual health services to improve the uptake of chlamydia and gonorrhea testing [10,11].

In England, public health commissioners are responsible for the process of planning, agreeing, purchasing, and monitoring health services [12]. The commissioning of free, open-access STI or BBV testing has primarily been the responsibility of local authorities since the implementation of the Health and Social Care Act 2012 [13]. Commissioners are responsible for identifying a preferred model of service provision and instigating a tendering process to select preferred sexual health services that can deliver the model within budgetary constraints. Although OPSS is increasingly part of sexual health service models, it has been adopted in different regions of England at different times, with different models of delivery. For example, OPSS is delivered by National Health Service (NHS) trusts in some areas and outsourced to sexual health clinical services, laboratories or online testing platforms provided by the private sector in others [14-17]. There is a lack of evidence as to why certain delivery models were decided on, how they have been implemented, or how implementation strategies have influenced the sustainability of OPSS into sexual health service delivery.

There is extensive evidence about factors influencing the adoption and embedding of digital health interventions in health care [18]. However, there are fewer studies that have considered the sustainability of their implementation, and this evidence gap has been noted specifically in relation to OPSS and other remote testing services for STI or BBVs [3,19]. In addition, it is unclear how applicable pre–COVID-19 findings (before March 2020) are to the current context of digital health implementation. This is important, given the impact of COVID-19 on normalizing online health care delivery [20].

### Objectives

This study is nested within the assessing the impact of OPSS for STIs on health inequalities, access to care and clinical outcomes in the United Kingdom (ASSIST) study, a comprehensive evaluation of OPSS within the UK context, that aims to assess the impact of these services on health inequalities, access to care, and clinical and economic outcomes and to identify the factors that influence the implementation and sustainability of these services [21]. This study focuses on the implementation of OPSS in 3 case study areas (CSAs) of England. In so doing, it addresses important evidence gaps in digital STI or BBV testing.

## Methods

### Design

Evaluation of OPSS implementation from staff and stakeholder perspectives formed 1 workstream within ASSIST, a wider mixed methods realist evaluation of OPSS, with the published protocol documenting the full study methods and design [21]. Separate papers covered the findings from other workstreams, for example, on service user experiences [22], health inequalities, access to care, and clinical and economic outcomes (Gibbs J, unpublished data, April 2025 and Jackson L, unpublished data, April 2025).

In this study, a combination of qualitative data collection methods were used: interviews, document analysis, and contextual observation. Methods and findings are reported in line with COREQ (Multimedia Appendix 1). Each data source had a distinct primary purpose and was initially analyzed separately, with preliminary findings from each compared, triangulated, and explanatory detail added where available. Details of implementation models, impacts, and sustainability were drawn primarily from interviews. Observations were used to understand how OPSS featured in clinic-based settings and to complement experiences of OPSS drawn from interviews. Documentary sources were primarily used to construct a timeline for implementation.

The design and methods were informed by normalization process theory (NPT), a substantive middle-range theory that has been used extensively for the study of the work required for initiating, integrating, and embedding (normalizing) digital health innovations into practice [23].

### Setting

We evaluated OPSS implementation in 3 CSAs in England, labeled CSA 1, CSA 2, and CSA 3 for anonymity. The CSAs were purposively selected based on them having undergone a distinct commissioning process for OPSS, with implementation at different times and the continuation of clinic-based services alongside OPSS. This enabled exploration of different delivery models at different stages following OPSS implementation. Each CSA was predominantly urban and had a population of at least 500,000, which was diverse in terms of socioeconomic circumstances and the proportion of people who are: ethnic minorities; lesbian, gay, bisexual, transgender, queer, and similar minorities; and young. These populations may be disproportionately affected by STIs or BBVs [21]. The focus of data collection was clinic-based and OPSS service settings, with 1 clinic-based service in each CSA chosen as the setting for contextual observation. These clinics were all level-3 sexual health services—offering the most complex care—and situated near the center of each CSA.

### Interviews

#### Sampling and Recruitment

We used a purposive snowball sampling strategy to reach a range of staff and stakeholders who could offer different perspectives on the implementation of OPSS. We sought to speak to people in leadership roles, such as commissioners, clinical leads, and service managers, who were involved in the decisions to adopt OPSS and staff at varying levels of seniority working in clinical and nonclinical roles in sexual health services (clinic-based and online) to understand the impact of OPSS on their work. We aimed to recruit up to 20 staff and stakeholders per CSA. Our sample size was determined by the concept of information power [24]. This enabled us to capture the range of diverse participant perspectives we were seeking, rather than to achieve data saturation, which would not have been feasible due to key perspectives—such as those of a commissioner—only being held by 1 person in some CSAs.

Primary investigators at each CSA were initially asked to circulate details of the study to staff working in their service, who contacted TS if they were willing to be interviewed. At the end of each interview, participants were asked to suggest other people they thought would be able to offer a useful perspective on the research topic. As interviews progressed across all 3 CSAs and we began to analyze the data, we noted roles that we had recruited in some CSAs but not others or that were likely to have a perspective on a topic that had arisen in other interviews. We then asked primary investigators to connect us with staff or stakeholders who could fill these gaps.

#### Data Collection

Semistructured interviews lasting 30 to 60 minutes were conducted, using Microsoft Teams, between January and December 2022. They each followed a topic guide covering: the adoption of OPSS; their perspectives of OPSS in relation to wider sexual health service delivery; the impact of OPSS on their and colleagues’ work; and their reflections on OPSS and its implementation, including its sustainability where relevant to the CSA (Multimedia Appendix 2; also available in the protocol by Gibbs et al [21]). Questions were tailored depending on whether participants were more involved in adoption or delivery. We mitigated for recall bias by seeking overlapping perspectives where possible and comparing interview data with contemporaneous documents. Participants were interviewed individually, aside from 3 with overlapping roles in CSA 1, who were interviewed together. All interviews were conducted by TS or JS.

#### Data Analysis

All interviews were audio-recorded, transcribed by a professional transcription company, and transcripts checked for accuracy. Our analysis began with NPT as a deductive framework to enable exploration of the normalization of OPSS within staff and stakeholder work (Multimedia Appendix 3) [23,25-27]. We also used the Consolidated Framework for Implementation Research (CFIR), which we felt was able to capture examples of implementation context more directly than NPT [28].

Coding was undertaken by TS and JS, using NVivo (Lumivero) software, who reviewed and refined the coding approach informed by discussion with the wider research team: FMB, JG, AH, GW, LJ, and DC. Interim findings were presented to staff at all research sites, including some who participated in the study and others who did not, to inform interpretation, check whether our interim findings aligned with their experiences of OPSS implementation, and verify that we had adequately captured the context in each site.

### Contextual Observation

#### Overview

TS conducted 2 forms of contextual observation in person at clinic-based sexual health services: think-aloud exercises with reception staff and nonparticipant observation of clinical consultations [29]. The aim was to obtain an understanding of how OPSS featured in practice, within staff work in clinic settings, to supplement interview accounts of perceptions and experiences. We were interested in both clinical staff who provide expert guidance to service users during consultations and reception staff who—we knew from the interviews—often interact with service users first and direct them toward OPSS or clinic-based services. As it would not have been feasible to obtain consent from service users before they presented at the clinic reception, we opted to use think-aloud exercises to explore how reception staff would interact with service users who might benefit from OPSS.

#### Data Collection

Reception staff present on the day of data collection were invited to take part in the research individually. They were each read 4 short scenarios about a service user who was presenting at the sexual health service (Multimedia Appendix 4). They were asked to say aloud what they were thinking about each scenario and how they would respond to the service user. TS would prompt them to keep thinking aloud and to explain which services they would direct service users toward and why. TS took contemporaneous notes as the participant was speaking.

Health advisors, whose role includes providing partner notification, management of people diagnosed with STI or BBVs, counseling, and signposting to services, obtained informed consent from any service users willing to have their consultation observed. TS would take notes during the consultation, primarily using a preagreed framework that focused on whether and how OPSS featured in each consultation, as well as what support staff were offering to service users that may be absent from an OPSS experience.

#### Data Analysis

TS took notes on whether and how OPSS was featured in spaces such as the waiting room or consultation rooms. Each site visit was discussed afterward by TS and JS to explore how it confirmed, contradicted, or provided further explanation to the interview data.

### Documents

#### Sampling and Data Collection

Documents were sourced in 3 phases. First, a Google (Google LLC) search was conducted to identify publicly available documents related to UK policy, strategy, and guidance on sexual health services and the role of digital platforms, limited to publications released after the Health and Social Care Act 2012. Search terms included “sexual health” plus “digital” or “online.” Sources of documentation included clinical guidelines, national strategy or policy, reports, position statements, or recommendations by the national government, government agencies, or clinical or service user organizations.

Second, specific searches were conducted to identify documents of relevance to the adoption and delivery of sexual health services in each CSA. Searches were conducted on Google and on specific local authority websites. The search was limited to documents published from 3 years before the delivery of OPSS in each CSA. Search terms included the case study region and areas within each region, with “sexual health” plus “digital” or “online.” Sources of documentation included needs assessments, meeting agendas and minutes, committee reports, newsletters, service plans, and strategies or reports of commissioning processes.

Third, we contacted each research site to seek documentation not in the public domain. We requested service specifications, strategy documents, and consultation documents as well as borough- and service-level minutes from meetings that recorded decisions around how OPSS was instigated, provided, and sustained. We also asked interview participants if they were aware of, and could share, documents that offered other insights into the implementation or delivery of OPSS in their CSA.

#### Data Analysis

Documents that did not mention online delivery of sexual health services were excluded. Included documents were cataloged by date, CSA (or national), and type. Documents were then ordered to build a timeline of implementation in each CSA by providing evidence of dates of when key developments in OPSS occurred.

### Data Synthesis

Interviews were the dominant source of data in our evaluation, and we first coded these sources using a coding framework based on NPT to analyze them. This initial analysis was used to develop early “pen portraits” of our CSAs and to inform analysis of documentary sources (ie, to identify pivotal contextual events or circumstances and the key implementation milestones in each site). Extraction from documentary sources was used primarily to provide the “hard” data on when key events happened and also, where available, to triangulate interviewees’ recollections.

As our analysis progressed, we inductively identified that the implementation of OPSS in all CSAs was part of—or took place alongside—substantial transformation of sexual health services. Therefore, we used other frameworks to inform our coding to complement NPT and added CFIR (Table 1), guided by the Dissemination and Implementation Models in Health webtool [26]. We applied a conceptual framework relating to major system change, developed by Fulop et al [27], to structure the account of how OPSS was conceived and implemented. We were also guided by the implementation outcome measures proposed by Proctor et al [30], which provided a structured framework for reporting implementation outcomes.

**Table 1:** Frameworks and theories used in the qualitative analysis of data (2015 to 2023) from staff and stakeholder interviews, observations and documents to understand the implementation of online postal self-sampling (OPSS) services in 3 case study areas (CSAs) in England.

Theory or framework	Key propositions	Application and adaptation to ASSIST^a^
NPT^b^ [31]	Normalization (embedding) of interventions requires work individually and collectively from all actors in the system. Normalization is not assumed to be a good or bad thing or permanent; processes can become denormalized.	Interview data collection: framed OPSS neutrally in interviews (ie, more implementation not presented as success). The topic guide was organized around the 4 implementation mechanisms in NPT: how actors made sense of OPSS and what benefits it might offer (coherence), how those leading implementation engaged others (cognitive participation), how all actors enacted it (collective action), and how all of them appraised its effects (reflexive monitoring). Analysis—deductive: initial coding framework based on NPT. Coherence aligned more closely with the implementation outcomes proposed by Proctor et al [30], as it was less of a determinant of implementation than a product of it. Data initially coded under collective action aligned better with the NPT coding manual relational restructuring [31].^d^
Implementation outcomes by Proctor et al [30]	Heuristic taxonomy of 8 implementation outcomes, distinguished from clinical and service outcomes: acceptability, adoption, appropriateness, feasibility, fidelity, implementation cost, penetration, and sustainability	Descriptive analysis—structuring framework: focused on 5 outcomes of most salience to ASSIST’s goals: acceptability, adoption, fidelity, penetration, and sustainability. (Cost is not covered as an outcome in this paper because it the focus of another ASSIST workstream; see Gibbs et al [21])
Major system change framework [27]	Identifies 5 pivotal points in the process from a decision to change to clinical outcomes	Descriptive analysis—structuring framework: provided structure to the account of OPSS implementation anchored to the first 4 stages of the framework: decision to change (aligned with the concept of adoption by Proctor et al [30]), model decided on, implementation approach, and implementation outcomes
CFIR^e^ [28]	Factors identified and described at inner and outer setting levels drawn from studies of determinants of implementation outcomes	Analysis—inductive: selected domains within CFIR inner and outer settings to provide more granular focus on contextual factors in ASSIST related to critical incidents, financing, and external pressures as key influences on implementation outcomes. As others have noted (eg, Nilsen and Bernhardsson [32]) it was not meaningful to apply CFIR’s distinction between inner and outer setting to our CSAs, given that OPSS was sometimes integrated with clinic services and sometimes separate

^a^ASSIST: assessing the impact of online postal self-sampling for sexually transmitted infections on health inequalities, access to care and clinical outcomes in the United Kingdom.

^b^NPT: normalization process theory.

^c^Italicization indicates how similar concepts featured in different frameworks were treated.

^d^CFIR: Consolidated Framework for Implementation Research.

We used these frameworks to develop a table that enabled us to make an initial comparison of the implementation in each CSA, from adoption to outcomes. We continuously refined the table categories and content as analysis progressed until we created the version and accompanying narrative presented in the results. Documents were used as a comparison with the interview data to explore where the documents confirmed, contradicted, or provided further explanation to the interview data. Contextual observation enabled us to test our findings derived from analyzing the interview data using the NPT framework, examining whether OPSS appeared in staff workflows as described in the interviews.

### Ethical Considerations

Ethics approval was granted by the NHS South Central—Berkshire B Research Ethics Committee (21/SC/0223). All participants provided written informed consent to be interviewed or observed as relevant to their role in the study. Data were deidentified by not naming the CSAs, removing any names from transcripts, and listing job categories rather than individual roles. Participants took part without compensation.

## Results

We interviewed 60 staff and stakeholders across our 3 CSAs, conducted 12 observations, and collated data from 86 documents (Table 2).

**Table 2:** Overview of staff and stakeholder interviews, observations, and documents (2012 to 2023) collected to understand the implementation of online postal self-sampling (OPSS) in 3 case study areas (CSAs) in England.

		CSA 1	CSA 2	CSA 3	Total
**Interviews**					
	Commissioners	3	6	1	10
	Senior managers and clinical leads	2	9	2	13
	Clinical staff (consultants, junior doctors, nurses, health advisors, and health promotors)	7	10	6	23
	Nonclinical staff (administrators, communications, and clinic managers)	3	3	2	8
	Stakeholders (pharmacists and external OPSS staff)	1	3	2	6
**Observations**					
	Observations: think-aloud exercises with administrative staff	5	0^a^	4	9
	Observations: clinics (patients)	1	1 (3)	1^b^ (3)	3
Documents (including needs assessments, commissioning specifications, tenders or service proposals, contracts, service guidelines, newsletters, user consultations, and case studies)		14	51	11	86 (including 10 national documents)

^a^The clinic-based service selected for contextual observation did not have the capacity to release staff to participate in the think-aloud exercises.

^b^Observations were of 1 clinic but 2 health advisors.

### Description and Comparison of Implementation Processes and Outcomes

#### Overview

We first provided a thematic description of the implementation process and outcomes, which are also summarized in Table 3. Further details are available in an extended data table, Multimedia Appendix 5. A timeline from the perspective of stakeholders in show, highlighting major contextual changes (blue), and implementation phases, with preparation in purple, OPSS delivery in pink and clinic changes in green. We then compared the influence of implementation strategies on implementation outcomes and their interaction with context.

**Table 3:** Description of online postal self-sampling (OPSS) implementation in 3 case study areas (CSAs), 2015 to 2023, drawn from the qualitative analysis of data (2012 to 2023) from staff and stakeholders, observations, and documents to understand the implementation of OPSS in 3 CSAs in England^a^.

			CSA 1		CSA 2		CSA 3
**Adoption decision**							
	Decision makers	Integrated sexual health service included proposal for OPSS within successful tender		Commissioners: commissioning process was for a standalone OPSS service		Commissioner: commissioning specifications for STI^b^ and BBV^c^ services included requirement for OPSS	
	Decision rationale	Aim to reduce the cost of delivering STI and BBV testing by shifting low-risk and asymptomatic service users from clinic-based services; reduce inequalities in access to STI testing; improve public health outcomes; give service users more autonomy; embed web-based or electronic pathways of care into the wider service		Aim to reduce cost of delivering STI testing by shifting service users from clinic-based services; reduce inequalities in access to STI and BBV testing		Aim to reduce inequalities in access to STI and BBV testing; reduce overall costs of STI and BBV services	
**Implementation approach**							
	Initial model of OPSS provision	In-house by sexual health service Unlimited testing Marketing campaign By post or collection at clinic, pharmacy, general practitioner, or charity		Outsourced—bespoke service Tests limited 4 times per year Limited marketing By post or collection at clinic		Outsourced—standard service Limited marketing By post or collection at clinic	
	Relationship with wider, clinic-based services	Part of wider service change (multiple to single sexual health service)		Parallel to wider service change (many to fewer sexual health services)		Followed wider service change (many to fewer sexual health services; contraceptive services separated from STI and BBV testing and contract awarded to a different organization)	
	**Implementation mechanisms**						
	Actions to engage all actors in implementation (cognitive participation)	A small task-and-finish group comprising members of the integrated sexual health service made informal decisions Extensive training and partnership days with staff to discuss transformation and delivery		Formalized process across 27 commissioning organizations at its launch in 2018 OPSS briefings to staff at each partner clinic		Sexual health service contracted OPSS OPSS briefings to staff at sexual health service	
	Timescales for launch	Rapid, launched 3 months after sexual health contract awarded		In stages, over 6 months (after 6-month delay)		Rapid, launched 2 months after sexual health contract awarded	
**Implementation outcomes**							
	Initial (pre–COVID-19) staff coherence and acceptability	Staff engaged with, and supportive of, implementation		Supported OPSS in principle, but some disengaged from, or were hostile to the implementation approach		Very little awareness of OPSS in the 2 months it was active pre–COVID-19	
	Penetration (initial; ie, speed and extent to which OPSS was delivered to service users)	Evidence from December 2015, 5 months after the contract was launched, reported a gradual increase in website visits and tests since the launch (before March 2020)^c^		OPSS testing delivered in phases across all sites, volume of testing increased month by month over 12 months (before March 2020)		Testing volumes much higher than anticipated (after March 2020)^d^ and difficult to control	
**Sustainability^d^**							
	Penetration (late—until end of ASSIST^f^ data collection 2022)	Since the lockdown in March 2020, OPSS testing delivery has reduced No collection in clinics and community during lockdown		Since the lockdown in March 2020, volumes of OPSS delivery have increased More commissioners signed up to model		—^e^	
	Fidelity and adaptations: unplanned	Delays in sending results, due to COVID-19 tests being prioritized by laboratories Challenges sourcing kit equipment Difficult to reset after the peak of the pandemic		—		—	
	Fidelity and adaptations: planned (reflexive monitoring)	Users limited to 1 order per month		Remit of OPSS expanded to those with mild symptoms and contacts of infection		Daily cap on OPSS orders introduced in 2022	
	Staff acceptability: staff work to enact and sustain OPSS (collective action and relational restructuring)	Staff remained supportive of OPSS Staff work changed: fielding inquiries about kits from patients and weekly meetings with laboratories and suppliers		Staff more engaged with OPSS Clinic staff work changed: increase in complex patients in clinic and no longer offer routine asymptomatic testing		Staff support OPSS in principle but see it as a separate service Staff engagement declined after the introduction of a cap on testingg	

^a^Evidence from data sources is provided in Multimedia Appendix 5.

^b^STI: sexually transmitted infection.

^c^BBV: blood-borne virus.

^d^March 2020 coincided with lockdown and closure or significant restrictions in clinic-based service delivery because of the COVID-19 pandemic (Figure 1).

^e^Not available.

^f^ASSIST: assessing the impact of online postal self-sampling for sexually transmitted infections on health inequalities, access to care and clinical outcomes in the United Kingdom.

^g^Most of ASSIST’s data collection was completed by the end of 2022, so sustainability in this paper relates to delivery until the end of 2022, but contextual observations and discussions with sites indicate that delivery has continued to change in 2023.

**Figure 1:**
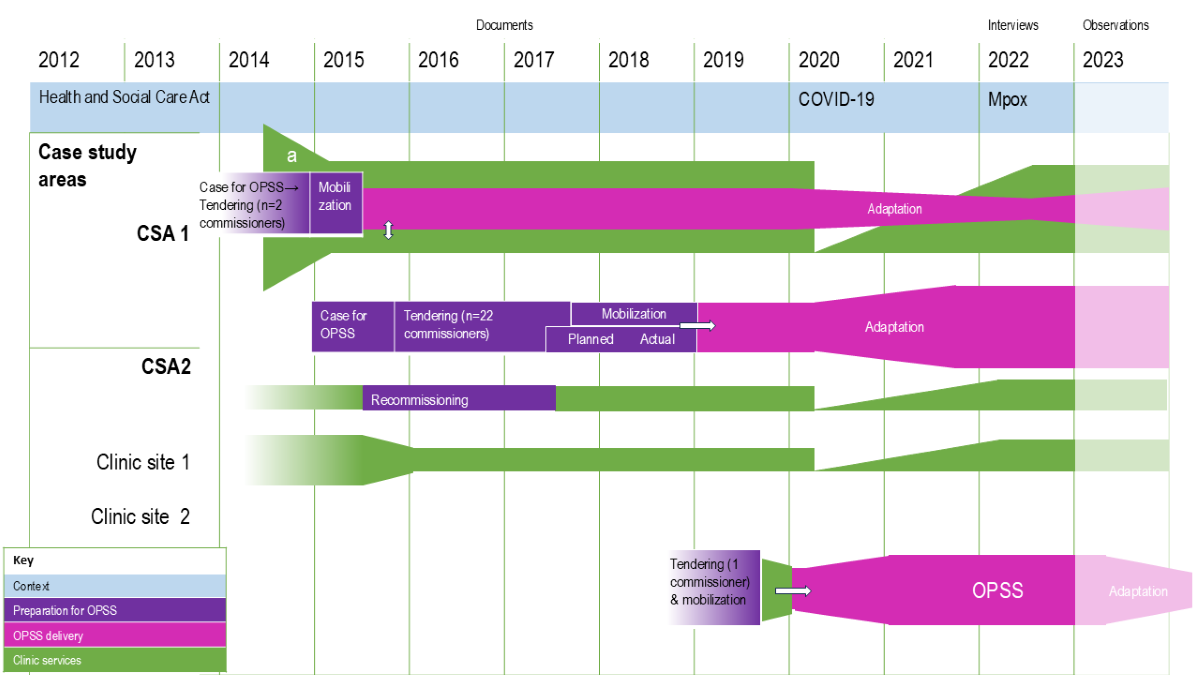
Timeline of adoption and implementation of online postal self-sampling (OPSS) services from analysis of documentary sources, interviews, and observations across 3 case study areas (CSAs) in England (2012 to 2023).

#### Adoption

In CSA 1, the decision to implement OPSS came from an NHS trust, which chose to include OPSS as part of its bid for a wider sexual health services contract. In contrast, commissioners initiated the decision to adopt OPSS in CSA 2, following a multiyear transformation program for sexual health, which recommended the introduction of a city-wide OPSS service. It was also initiated by commissioners in CSA 3, who specified that OPSS needed to be part of the delivery model.

#### Implementation Approach

CSA 1 started delivery within 3 months of the trust being awarded the new, integrated sexual health contract covering the entire city and a neighboring local authority. Its implementation was very rapid, with cognitive participation (ie, the relational work to build and sustain OPSS) driven by a small in-house team, often working informally. CSA 1’s in-house model saw every aspect of the service—the website, pathology, and results communication—delivered by the NHS trust except kit production and dispatch, which were outsourced. It integrated OPSS with other face-to-face services, which were equipped to disseminate kits and receive them with completed samples—an example of collective action (ie, the work done to implement and sustain OPSS). The service was launched with a marketing campaign to encourage uptake. There were few restrictions on who could access OPSS or how often.

CSA 2, in contrast, was rolled out in phases, starting with 1 to 2 areas in January 2018, then in all commissioned areas by June 2018. The implementation of OPSS took place alongside other transformations that were commissioned separately, including the integration of sexual health and contraceptive services and some clinic closures. OPSS was not widely advertised—the aim being to shift existing service users from clinic-based testing to internet-based testing rather than increase overall testing.

The sexual health service in CSA 3 put out a commissioning specification for an OPSS service after it had commenced the new sexual health contract. OPSS was launched 2 months later. The new contract separated sexual health from contraceptive services, which were delivered by a separate organization.

CSAs 2 and 3 outsourced OPSS to a private company; CSA 2 commissioned a bespoke service, while CSA 3 bought in a standard service. Kits in both areas were predominantly posted, although some were collected at clinics. Marketing was limited, primarily to clinics. Patients could only access 1 kit every 3 months and needed to be asymptomatic.

#### Acceptability of OPSS

We found the strongest and most consistent support for OPSS among staff in CSA 1. There was a coherent view that embedding digital services would improve access and ensure patients with greater need could be seen more easily in clinics. Staff felt engaged in the implementation process and engaged with service users regularly about OPSS, although it increased work in some circumstances, such as when service user data were split between the electronic patient record systems of the NHS trust and the pharmacies that had been contracted to deliver and receive OPSS kits.

In contrast, there was a lack of shared coherence among stakeholders in CSA 2. Commissioners stated that their aim was to maintain STI or BBV testing amid rising demand and reduced funding; however, they also hoped that OPSS would improve access and public health outcomes. Clinic staff, in contrast, often felt that commissioners prioritized cost savings at the expense of meeting service user needs. Staff were initially unaware of, and were not engaged with, OPSS implementation, and some were openly hostile to its implementation.

There was support for OPSS in principle among CSA 3, but the sexual health service expressed concerns about the lack of control over volumes of testing demand, which was much higher than anticipated, and the ensuing spiraling of costs.

#### Sustainability

CSA 1’s service was severely disrupted from March 2020 by the COVID-19 pandemic, when laboratory capacity within the trust was redirected to testing for COVID-19 and there were challenges sourcing equipment for OPSS kits. Provision of kits decreased, and the kit processing times increased. This required new collective action, with many staff having to deal with service user inquiries about delays to receiving kits or results on a regular basis and some engaging in work to mitigate the disruption, such as weekly meetings with the laboratories and kit suppliers. Kit orders were restricted for the first time to 1 per month. However, staff acceptability in CSA 1 remained high, despite these challenges in delivering OPSS.

In CSA 2, delivery of OPSS increased during the COVID-19 pandemic, and—alongside remote consultations—it enabled STI or BBV testing and treatment to be maintained amid restricted access to face-to-face clinic appointments during lockdowns. The remit of OPSS was expanded to include users with mild symptoms and those whose partners had tested positive for an STI or BBV. Many clinics continued to redirect all asymptomatic service users to OPSS following the end of pandemic restrictions. OPSS was also used to manage demand during the mpox epidemic in 2022, which placed additional pressure on clinic-based services. Staff reported being more aware of OPSS after the pandemic and participating in more collective action, referring service users to OPSS more often and accessing results more frequently, although this did create additional work, such as having to access multiple electronic patient record systems. Clinic staff noted that while OPSS reduced visits from asymptomatic service users, it also brought changes to their clinic workflows. For health care support workers, who had previously managed in-person testing for asymptomatic service users, their role became more administrative, and staff noted that it reduced career development opportunities for them. In contrast, more senior clinical staff saw an increased complexity in their clinic caseloads, and this led to the instigation of longer consultation slots in some settings.

In CSA 3, use increased far higher and more rapidly than anticipated, largely because OPSS was launched shortly before the pandemic. This was challenging to reset after pandemic restrictions ended. Reflexive monitoring required additional work by clinic management and external stakeholders to explore ways to reduce the cost of delivering OPSS. The commissioner provided additional funding to meet this demand, and eventually, a cap on daily orders was agreed upon by commissioners and the sexual health service, making clinics once again the primary access point for testing. In our interviews conducted 2 years after OPSS had been introduced, there was still a lack of coherence among staff in CSA 3, with many believing that OPSS had been introduced in response to the COVID-19 pandemic. Many also believed that it was an entirely separate service, rather than being commissioned and clinically led by the trust. Staff saw value in OPSS to improve access, but there was a strong consensus via reflexive monitoring that it was too expensive to deliver.

### Explanations of Implementation Variation

Two contextual factors determined the implementation approaches available to service leaders and had a substantial impact on the implementation outcomes in all CSAs. The influence of each factor was further impacted by a third contextual factor, the COVID-19 pandemic.

#### Decreasing Budgets for Sexual Health Services

Participants involved in the decision to adopt OPSS frequently noted that decisions were made because of, or with reference to, reductions in budgets for STI or BBV testing. The pressure on budgets was noted as particularly acute following the transfer of responsibility for most sexual health service commissioning to local authorities. Its stated importance as a driver to implement OPSS was different across CSAs, and there was a lack of consensus within some CSAs on the extent to which it affected OPSS decisions.

Staff in CSA 1, which launched before the steepest decline in national sexual health budgets, told us that while the decision to adopt OPSS was partly driven by a desire to reduce the cost of service delivery, there was also a very strong emphasis on reforming services to improve access, embed digital, and reduce health inequalities:

The [local authority] Director of Public Health at that point was very proactive...and clearly wanted to change how we delivered this service, particularly around decentralisation and a more equitable service I think. More opportunities for people to access it.

Participant 38, clinical, CSA 1

The commissioner in CSA 3—who took the decision to adopt OPSS—similarly saw the introduction of OPSS as an opportunity to deliver better outcomes amid funding constraints:

There are areas on the outskirts of the city which are 16 miles away from a city-centre clinic so I felt quite strongly that we- although we had a good service we had an inequitable service.... At the same time we’d also [had] five years of cuts to the public health grant that resulted in us closing the city-centre contraception clinic so again that compromised access even further. So those factors really led me to a place of believing that if we needed to improve the service and improve access to STI and contraception care that it needed to be redesigned.

Participant 50, commissioner, CSA 3

Commissioners in CSA 2, by contrast, noted their main reason for introducing OPSS was to maintain access amid rising demand and reducing income, although they also hoped to increase access and public health outcomes:

Because of the financial trouble that authorities were in, with the cuts to the public health grant, STI testing had been going up and up in CSA2 and then—for two years before [the OPSS service launched]—stopped because the councils ran out of the money and they needed a way to grow activity without growing costs at the same rate.

Participant 15, commissioner, CSA 2

The financial landscape in turn shaped the delivery model and implementation approach in each CSA. CSA 1 chose to deliver OPSS in-house, enabled by the trust’s ability to fund the required infrastructure:

...doing it internally was better and the reasons for that were mostly integration and cost. Because clearly we want the people who are tested on like postal testing to have their results all in our own system rather than having it imported later. And also looking at the costs actually the external costs were very high compared with what we could do internally.... The reason it happened primarily was there was senior management buy in...the agreement to invest some money into it and the driver of losing the service essentially unless we could.

Participant 38, clinical, CSA 1

CSA 1 also took a proactive approach in marketing OPSS to encourage uptake. This approach was resisted in CSA 2 to avoid placing more demand on the sexual health budget. Similarly, CSA 3 stated during its bid for the contract that OPSS use would need to be constrained for the service to be financially sustainable:

The level of online testing and future trends would be closely monitored to ensure negative consequences of burgeoning demand are avoided, i.e. reduced positivity and the potential to significantly exceed the financial envelope.

Document S7, contract, CSA 3

This budgetary context also shaped implementation outcomes, such as staff acceptability. Clinic staff in CSA 2, for example, were more likely to see OPSS as a cost-saving measure rather than a benefit to their service and had more concerns than staff in other areas about the risk OPSS posed to their services and jobs. In CSA 3, where the costs of OPSS were directly drawing funding from clinic-based services, there was more stated concern about affordability:

I think at the end of the day it’s all about money, you know, which I think’s rather sad because when I look at my own Trust...we had lots of lovely small satellite services dotted all over [the area]. We’ve had to shut them all because again the asymptomatic pathway is all being channelled through [OPSS].

Participant 5, nonclinical, CSA 2

Staff in CSA 1, in contrast, were far more positive about the value OPSS brought to their service, despite some service closures and job losses during the commissioning process:

I think back when we first started yes, we was all quite positive about it. You know it took the pressure off the clinics and the walk-in clinics we used to do for people who would just walk-in and just want a general test. We could say you know rather than you sitting waiting for a few hours to be seen why don’t you do this and they were quite grateful of it a lot of them were.

Participant 32, nonclinical, CSA 1

#### Organizational Structure, Culture, and Relationships

The relationships between organizations involved in the commissioning and delivery of OPSS, the division of work between these organizations, and the culture within them were all strong influences on implementation outcomes.

CSA 1’s decision to deliver its OPSS almost entirely within the trust, for example, rather than outsourcing it to an external organization, allowed it to launch very quickly:

We decided what we were going to do and we up and did it, I’ve nothing written down at all, there were no minutes taken in meetings, we acted quickly with it and because we were all in the same [organization].

Participant 38, clinical, CSA 1

However, the informal relationship between stakeholders within the trust, such as the sexual health service and the pathology department, meant that equipment could be redirected away from STI and BBV testing and toward COVID-19 during the pandemic:

Covid just killed our online testing service.... So Covid started and we’re using the online testing service and all of a sudden...our main supplier cannot supply us the components simply because there was this competition between them supplying the same kits for Covid testing and STI kit.... At that time it was the height of the pandemic...it’s sexual health, no, we’re not important. Everything, all the efforts went into Covid. So that was a massive challenge to a point that we have to completely switch off our self-testing kit website and change our guidelines.

Participant 34, clinical, CSA 1

CSAs 2 and 3, in contrast, were able to expand OPSS delivery in 2020 because of outsourcing it to a specialist company. However, in CSA 3, there was a much higher unit cost than CSA 2, which was able to commission at scale as a consortium of local authorities. In addition, the sexual health service in CSA 3 was not able to make a unilateral decision to cap testing—it had to liaise with the local authority commissioner to make changes to the contract:

So when the contract came out, it asked us to upscale online testing over a period of five years from very little to 30% of our screening total and it very quickly obviously because the pandemic was forcing to almost 70-80% and reeling that back now is extremely difficult because the only way to reel it back in that isn’t extremely complicated is to cap testing. Most commissioners are a bit reluctant plus it is also a blunt tool because you don’t know who you are capping.

Participant 48, clinical, CSA 3

The number of local authorities and sexual health services involved in CSA 2’s commissioning process meant that relationships were highly formalized and there was a lack of coherence between some stakeholders. This contributed to an antagonistic culture among some:

[Meetings with commissioners] would be in seminar rooms...so you are sitting across each other and we need you to do X, Y, Z, we think this is that...it’s kind of like, well no I don’t agree...we were defensive of our service and what we needed to do and defensive of our population as well...to have a blanket approach and blanket targets we didn’t agree with. And so they listened but they were all defensive and they had their own agenda about reshaping services and reducing cost.

Participant 2, clinical, CSA 2

Some people saw part of what they do being taken away from them, is my sense, and felt very threatened about that like what is their purpose, their value, their specialness? And saw it is a direct kind of challenge and threat to that because...they have been indoctrinated and trained into a need to keep people passive and depending on them. Because it maintains their power and specialness.

Participant 15, commissioner, CSA 2

It also may have contributed to the longer, 3-year process of agreeing on the decision to commission OPSS and for it to be operational, as opposed to 6 to 12 months in the other 2 CSAs (Figure 1).

## Discussion

### Principal Findings

This study of the implementation of OPSS for STI or BBVs across 3 diverse CSAs found that budgetary pressures and organizational relationships determined the implementation strategies available to decision makers and how these strategies were enacted. Our study additionally illustrates the profound but divergent effects the COVID-19 pandemic had on OPSS implementation in different contexts.

### Strengths and Limitations

Our study has several strengths. Its multisite design allowed comparison of implementation strategies, and our combination of documentary sources over 12 years, with contemporary interviews and observations, enabled us to examine outcomes, including sustainability. Combining interviews with documentary sources and contextual observations provided richer insights than interviews alone. For example, the observations generated a more nuanced understanding of the collective action in “the real world,” where OPSS was less dominant than in an interview specifically about OPSS implementation. It also provided unintended insights into sustainability, for example, how CSA 3 implemented a daily cap on OPSS testing. Our use of multiple theoretical frameworks allowed these to complement each other and develop a more comprehensive understanding of implementation outcomes—and the factors that led to them—at different levels. The major system change framework helped to standardize the narrative across the 3 CSAs, enabling us to compare implementation approaches and outcomes, while NPT was particularly helpful for surfacing the implications on staff work (captured under “collective action”). CFIR enabled us to capture wider contextual factors, such as financing, policies, and laws, that influenced our findings using the other 2 frameworks.

Our study faced some limitations. The CSAs in this study were very different in size and complexity. On the one hand, this was a strength of the study in surfacing contrasting implementation approaches and outcomes within the same sexual health care system. On the other hand, it affected the research processes we were able to undertake. For example, we were not able to source key documents from all CSAs, such as tender specification and contracts. This was in part because nearly 10 years had elapsed since OPSS had launched in 1 CSA, and staff turnover meant no one in post still had access to documentation from the time of adoption. This was partly due to different implementation approaches; those that followed more formal processes had extensive documentation, whereas others documented fewer aspects. This made it harder to ascertain certain aspects of implementation and compare between CSAs and to corroborate some of the interview data. The time elapsed since the launch of OPSS in some CSAs may have introduced recall bias in certain interviews. In addition, some of the study team worked for, or closely with, sexual health services included in our evaluation. However, the data collection and analysis were conducted by researchers who did not have these relationships.

### Comparison With Prior Work

The coherence of OPSS to staff in this study aligns to some extent with the findings of other studies. In their review of home-based sexual health care, Goense et al [3] reported that staff believed “home-based STI testing” could improve access, especially for clients vulnerable to STIs. The interview study staff in the study by Chabot et al [33] articulated an expectation that home-based STI testing would provide increased convenience for users. However, there was less evidence of staff conflict or a lack of coherence in these studies than we identified in our study. The research by Ryu et al [34] on the experiences of sexual health services during COVID-19 lockdowns in Ontario in Canada did identify some concerns among staff. They were primarily concerned about the risks to service users not having sufficient privacy at home to access services remotely and staff finding it difficult to provide emotional support and education outside of face-to-face interactions. While staff concerns in this study did include risks to service users, the ambivalence or opposition to OPSS in some CSAs may have been driven by wider sexual health transformation changes, which also affected staff employment and service viability [35].

Ryu et al [34] identified that the pandemic catalyzed the adoption and implementation of remote models of sexual health care, which persisted beyond the pandemic. This aligns to some extent with experiences in 1 of the 3 CSAs in our evaluation, where expanding the scope of OPSS improved its acceptability among staff in CSA 2. However, our findings illustrate how COVID-19 could have contrasting effects. It severely undermined OPSS in CSAs 1 and 3, although for different reasons. The interaction of the organizational context and OPSS implementation approaches helps us to understand why the pandemic had such contrasting effects. These findings contribute toward addressing an evidence gap articulated by Goense et al [3] by providing insights on what affects the sustainability or maintenance of OPSS services. They also address a wider gap in implementation science literature, where context has been well established as an important factor in implementation, but there is limited evidence on how it can dynamically affect implementation approaches, particularly over time, or its relationship with implementation outcomes [19,25,28,32].

Existing research has observed that financial resources are one of the most commonly cited contextual factors affecting implementation, and as Nilsen et al [32] note, implementation research so far has focused predominantly on the need for sufficient financial resources to enable implementation to happen [36]. However, our study affirms a contrasting perspective, in that OPSS was implemented precisely because of concerns about insufficient financial resources [37]. Commissioners and services appeared to be faced with an unenviable choice: implement this “new, untested” service or face a failure of sexual health services altogether [38]. This distinct context, where implementation is initiated because of financial pressures, may help explain some of the surprising findings of this study and could have implications for other digital health innovations. For example, NPT posits that embedding of an innovation is more likely when staff perceive its benefits [23]. In this study, clinic staff acceptability did not predict implementation success; instead, implementation success—or otherwise—influenced acceptability.

We initially approached OPSS as a digital health innovation and sought to evaluate it as such. However, our findings suggest that implementation of OPSS was part of a process of large-scale service transformation [39] and as such has more in common with digital transformation, in that it involved several organizations and was associated with changes in funding and staffing models across both clinic-based and internet-based services. The rapid pace of this transformation in 2 of our CSAs—which each implemented OPSS within 6 to 12 months—offers insight into why they faced challenges with sustainability, especially when compared to the more gradual implementation in CSA 2 and other digital sexual health innovations internationally, such as the 3-year implementation of Get Checked Online in British Columbia in Canada [40]. This rapidity may have hindered the capacity to obtain sufficient local evidence to effectively guide implementation and suggests that future studies of the implementation of OPSS or similar intentions should consider, as Chabot et al [33] have done, how OPSS is embedded in the wider social and structural processes of the public health system.

### Conclusions

In this multisite case study, OPSS implementation was part of systems change to address a wider problem of insufficient funding to deliver sexual health care. This systems change, alongside organizational relationships, determined the implementation strategies available to decision makers and the outcomes they achieved, as evidenced by stark differences in sustainability during COVID-19. Implementation of OPSS may have progressed before sufficient evidence to effectively guide implementation was available.

On the basis of our findings, we provide 3 actionable recommendations for implementing OPSS that could contribute to successful and sustainable implementation of OPSS services into sexual health systems.

First, those planning to introduce OPSS should allow sufficient time in the preparatory phase to iterate plans, informed by stakeholder feedback, to build coherence among staff and service users. Our study documented that the process of introducing OPSS varied significantly between CSAs in terms of timelines, stakeholder engagement, and how formalized the process was. Our findings suggested that very rapid implementation may affect later sustainability and that local authorities commissioning as collectives are able to deliver OPSS at lower cost, particularly when outsourcing. Sexual health system leaders considering introducing OPSS would benefit from considering the implications of their business and delivery models before implementation.

Second, professionals delivering OPSS and those commissioning OPSS should build flexibility into implementation so that delivery and models can be altered to respond to changes in context. Our findings illustrate how implementation of OPSS is not a one-off event but an ongoing process that is sensitive to the context into which it is introduced and sustained. In our CSAs, important contextual issues included public service budget cuts, the sexual health service transformation agenda, and the COVID-19 pandemic, which affected the process and outcomes of implementation, particularly CSAs’ capacity to respond to changes in demand for OPSS. The nature of contextual changes may not be predictable, but building in the capacity to increase or reduce delivery of OPSS—and rebuilding staff coherence around any changes—could enhance sustainability of implementation.

Third, professionals delivering OPSS and those commissioning OPSS should reflexively monitor changes to staff workload across the sexual health system following introduction to OPSS. Our study found that introducing OPSS into sexual health systems changes the work of different staff groups in heterogeneous ways. Some of these changes could not have been anticipated, but continually monitoring and meaningfully responding to such changes could enhance the acceptability of OPSS to staff, enhancing its adoption and sustainability.

## Appendix

Multimedia Appendix 1 Consolidated Criteria for Reporting Qualitative Research (COREQ) checklist.

Multimedia Appendix 2 Topic guide, tailored to different interviewees in the study.

Multimedia Appendix 3 Coding frame based on the normalization process theory codebook (May 2022).

Multimedia Appendix 4 Observations and think-aloud data collection tools.

Multimedia Appendix 5 Extended data table.

## Abbreviations

ASSIST: assessing the impact of online postal self-sampling for sexually transmitted infections on health inequalities, access to care and clinical outcomes in the United Kingdom
BBV: blood-borne virus
CFIR: Consolidated Framework for Implementation Research
CSA: case study area
NHS: National Health Service
NPT: normalization process theory
OPSS: online postal self-sampling
STI: sexually transmitted infection

## Data Availability

The datasets generated or analyzed during this study are not publicly available due to public availability not being part of participant consent.
