# Supplementary material for: Evaluating the Implementation of Online Postal Self-Sampling for Sexually Transmitted Infections in England: Multisite Qualitative Study
Source: J Med Internet Res. 2025 Sep 9;27:e72812. doi: 10.2196/72812 (PMC12457857; doi:10.2196/72812)
Supplement: Multimedia Appendix 2 [file jmir_v27i1e72812_app2.docx]

**Digital health intervention or major system change? A multi-site qualitative study of the implementation of online postal self-sampling (OPSS) for sexually transmitted infections (STIs) in England**

**Additional file 1.**

**Implementation health care professional (delivery) topic guide**

| 1. **YOU, AND YOUR ROLE**   Before we start the interview, could you describe your role(s) in the service?  *[NB we’re expecting their role to be purely clinical or administrative but if they have a role in management, have handy the WS 3 topic guide to ask relevant questions on how they adopted OPSS]* |
| --- |
| 1. **INTRODUCTION OF OPSS /FIT WITH EXISTING VALUES AND ETHOS**  - When did you start working for [sexual health service]?   - (If relevant) When did you **first hear** about the possibility of using OPSS in this service? How was OPSS first introduced to you? - What did you think about OPSS when you first heard about it? *Prompt after each thing they think about OPSS: What makes you say that?* - (If present when OPSS introduced) What did your colleagues think about OPSS? Why did they think that?   *Prompt for range of views amongst colleagues; explore if particular professional groups had particular reactions.*   - What, in your opinion, influenced the **decision to introduce OPSS** in your service/area?   *Possible prompts if an interviewee goes silent after the first two questions:*  *external: e.g. covid– pre 2020 - LT plan & role of technology in healthcare, alignment with regional strategy (e.g. pan London)*  *organisational/local – service priorities or recognised problems, e.g. capacity constraints for f-f testing, budgets,*   - How does it fit with the existing ethos/culture of your service?   *Possible prompts: e.g. attitudes towards adoption of new technologies, experience of innovation and change, leadership support/informal or formal e.g. champions* |
| 1. **UNDERSTANDING OF OPSS IN YOUR CONTEXT**  - Could you describe for me how remote testing and treatment is delivered in your area? Possible prompts: what technology are you using? [e.g. online platform, website], in what part of your service? For which client groups? How do they access it? - How does this differ from what you did before? [explore both pre-lockdown and during lockdown periods] - When is OPSS recommended in your service/area? Why? Could you talk us through a typical pathway for OPSS vs clinic-based (both face to face and remote) service provision? - How do you feel about OPSS becoming the dominant testing pathway since Covid? What would you ideally like to see happen when the pandemic ends? |
| 1. **IMPACTS OF OPSS ON YOU, SERVICE, TEAM**   Can we turn now to the impact that OPSS has had on your work, and the work of colleagues.  How has OPSS changed your **interactions with patients / consultations**? How does that feel?  *Prompts: Effects on ease and effectiveness of consultations?*  *(if administrative role then* How has OPSS changed your **interactions with other clinic staff**? How does that feel *?)*   - Which do **you** prefer: face-to-face vs remote (invite comments on both telephone and online video consultation)? Why? - Are there different people you think each option works ‘better’ for (or not)? Why?   What **processes to support you to** **deliver** OPSS were put in place? E.g. training, new equipment   - How useful to you was all of this? Why?   In your opinion how do you think OPSS changes the **skills** you need to do your job? If so, how, and how does that feel?   - Did you, or your colleagues, need training before you could use OPSS? Did you receive any training and was it appropriate? How hard / easy did you find it to learn to use the system? - Conversely are there skills that you used to use before OPSS that you don’t need (or need as much) since OPSS?   Can you talk me through an example of something that you personally do differently since the introduction of OPSS?   - Has OPSS had any impact on the **division of work or the relationships between different professional groups** working in sexual health services? (e.g. nurses and doctors)? If so, how, and why?   Is there anything special or different about **the service you work in** that made implementing OPSS particularly hard or easy? If so, what / why?  *Possible prompts:*   - *staff structure* - *service priorities or recognised problems, e.g. capacity constraints for f-f testing, budgets,* - *ethos/culture* - *. attitudes towards adoption of new technologies, experience of innovation and change, leadership support/informal or formal e.g. champions*   Since the COVID-19 pandemic, a lot of health services had to do things remotely. Has this changed your views of OPSS? Why? |
| 1. **REFLECTIONS AND CHANGES**   Now that you’ve been using OPSS for a while…   - What **changes have been made** since you started? And/or are there any changes you’ve made personally?   *Can you explain why they were made?*  *Would like to make further changes? Why is that?*   - Some people have mentioned **changes to the context of delivering OPSS** since you have started using it. What do you think has changed? How have these changes affected OPSS in your service? *Possible prompts:*   - *External – Covid (as an enabler/accelerator of change?)*   - *Organisational/system level – reagent, lab capacity restrictions*   - *Local - staff redeployment in team* - What aspects of OPSS have **worked well**?   *Prompts: For you, personally? The way the service works? For patients?*  *Prompts: explore why they felt it worked well*  What do you think **could have been/should be done better**?  *Prompts: For you, personally? The way the service works? For patients?*  On reflection, **is it/has it been worth the hard work**? Why? |
| 1. **CLOSING**   We’re reaching the end of the interview.  Is there **anything else** you would like to raise?  What other information do you think would be useful to us to understand implementation of OPSS in your service?  Probes   - Who would you recommend we interview? - What documents would you suggest we read to help us understand the implementation processes? Eg protocols, care pathways? Business cases? |

**Implementation stakeholder & staff (adoption) topic guide**

*Tailor questions asked according to:*

- *Role in the service. Some indications given for questions most relevant to a) those involved in the decision to adopt OPSS and devising implementation strategies and b) those who had no role in the decision but are required to adapt their practice to OPSS. Note: for some HCPs both types of questions are relevant.*
- *Stage of implementation – initiation (ie at the decision to adopt), adoption (work is mainly focused on getting the practice into the service) embedding (OPSS being used but focus is on making it routine).*

*Tailoring question:*

| **YOU, AND YOUR ROLE**  **Before we start the interview, could you describe your role(s) in the service?**  **Interviewer: use this information to decide whether to use questions for**   1. **Lead clinician/commissioner that made the decision to adopt OPSS (ref. Protocol 3.1)** 2. **Those involved in decisions about how to adopt & implementation processes +/- role in delivery (ref protocol 3.2)** 3. ***[NB Many will also have clinical or administrative roles and hence also direct or indirect involvement in the delivery of OPSS, so have handy the WS 1.2 topic guide to ask relevant questions on how they deliver OPSS]*** |
| --- |

1. **UNDERSTANDING OF OPSS IN YOUR CONTEXT**

- **Could I check what stage OPSS is at in your area? (*initiation, adoption, embedding*)**
- **Could you describe for me how OPSS is [or will be] delivered in your area? *Possible prompts: what technology are you using? e.g. online platform, website, in what part of your service? For which client groups? How do they access it?***

| ***A. LEAD CLINICIAN /COMMISSIONER***   1. **INTRODUCTION OF OPSS /FIT WITH EXISTING VALUES AND ETHOS**  - **Can you talk me through how OPSS came to be delivered in your area?**   - **When did you first consider the possibility of using OPSS in this service?**   - **What were the stages followed in its introduction?** - **What did you think about OPSS when you first heard of it? *Prompt after each thing they think about OPSS: What makes you say that?*** - **(If relevant) How had your view changed by the time you started discussing introducing OPSS in your area? Why?** - **(If relevant) How did you introduce OPSS to others involved in the decision?** - **What did your colleagues think about OPSS? Why did they think that?**   ***Prompt for range of views amongst colleagues; explore if particular professional groups had particular reactions.***   - **What influenced your decision to introduce OPSS in your service/area?**   ***Possible prompts if an interviewee goes silent after the first two questions:***  ***external: e.g. covid– pre 2020 - LT plan & role of technology in healthcare, alignment with regional strategy (e.g. pan London)***  ***organisational/local – service priorities or recognised problems, e.g. capacity constraints for f-f testing, budgets*** | **B*.THOSE INVOLVED IN DECISIONS ABOUT HOW TO ADOPT***  **2. INTRODUCTION OF OPSS /FIT WITH EXISTING VALUES AND ETHOS**   - **When did you first hear about the possibility of using OPSS in this service? How was OPSS first introduced to you?** - **What did you think about OPSS? *Prompt after each thing they think about OPSS: What makes you say that?*** - **What about your colleagues? What did they think about OPSS? Why did they think that?**   ***Prompt for range of views amongst colleagues; explore if particular professional groups had particular reactions.***   - **What, in your opinion, influenced the decision to introduce OPSS in your service/area?**   ***Possible prompts if an interviewee goes silent after the first two questions:***  ***external: e.g. covid– pre 2020 - LT plan & role of technology in healthcare, alignment with regional strategy (e.g. pan London)***  ***organisational/local – service priorities or recognised problems, e.g. capacity constraints for f-f testing, budgets,*** |
| --- | --- |
| - **How does the use of OPSS in your service differ from what you did before? *[explore both pre-lockdown and during lockdown periods]*** - **How does OPSS fit with the existing ethos/culture of your service?**   ***Possible prompts: e.g. attitudes towards adoption of new technologies, experience of innovation and change, leadership support/informal or formal e.g. champions***   - **What did you do in your service to [start planning for/introduce] OPSS?**   ***Possible prompts: who was/is on a working group if it existed? Who was involved in getting approvals, awareness raising, protocols?*** | |
| 1. **IMPACTS OF OPSS ON YOU, SERVICE, TEAM**   **Can we turn now to the process of implementation and the impact that OPSS may have had on clinical work.**  **What processes [did you/were] put in place to support staff to deliver OPSS*? E.g. training, new equipment, change in roles/clinical pathways? How useful did you think they were. Why?***  **In your opinion how do you think OPSS changes the skills that staff in the service need to do their job?**  **Has OPSS had any impact on the division of work or the relationships between different groups working in sexual health services? (e.g. nurses and doctors and administrators)?**  **Is there anything special or different about the service that made implementing OPSS particularly hard or easy? If so, what / why?**  ***Possible prompts:***   - ***staff structure*** - ***service priorities or recognised problems, e.g. capacity constraints for f-f testing, budgets,*** - ***ethos/culture*** - ***attitudes towards adoption of new technologies, experience of innovation and change, leadership support/informal or formal e.g. champions***   **With the COVID-19 Pandemic, a lot of sexual health services had to do things remotely. Has having to do this changed your views of OPSS? Why?**  **How likely do you think it is that the changes which came about as a result of the COVID-19 pandemic (especially the increased use of OPSS) will be permanent? Why? How do you feel about this?**  **How has OPSS changed interactions with patients / consultations?**  ***Prompts: Effects on ease and effectiveness of consultations?*** | |
| 1. **REFLECTIONS AND CHANGES**   **Now that you’ve been using OPSS for a while…**   - **What changes have been made since you started? Can you talk us through these changes and explain why they were made? Why? Would like to make further changes? Why is that?** - **Some people have mentioned changes to the context of delivering OPSS since you have started using it. What do you think has changed? How have these changes affected OPSS in your service? *Possible prompts:*** - ***External – e.g. Covid (as an enabler/accelerator of change?)*** - ***Organisational/system level – e.g. reagent, lab capacity restrictions*** - ***Local – e.g. staff redeployment in team*** - **What do you see as the main advantages and disadvantages of face-to-face vs remote sexual health care delivery? Why?**   **In your opinion are there different clients it works ‘better’ for (or not)? Why?**  **What aspects of OPSS have worked well?**  **Prompts: For you, personally? The way the service works? For patients?**  **Prompts: explore why they felt it worked well**  **What do you think could have been/should be done better?**  **On reflection, is it/has it been worth the hard work? Why?** | |
| 1. **CLOSING**   **We’re reaching the end of the interview.**  **Is there anything else you would like to raise?**  **What other information do you think would be useful to us to understand implementation of OPSS in your service?**  **Probes**   - **Who would you recommend we interview?** - **What documents would you suggest we read to help us understand the decision making and implementation processes? Eg protocols, care pathways? Business cases?** | |

**Thank you**

**Thank you so much for taking the time to talk to me today – your views are really helpful for our research. If you are interested, we will be in touch with the findings from our research. In the meantime, please do get in touch with any questions.**
