# Supplementary material for: Evaluating the Implementation of Online Postal Self-Sampling for Sexually Transmitted Infections in England: Multisite Qualitative Study
Source: J Med Internet Res. 2025 Sep 9;27:e72812. doi: 10.2196/72812 (PMC12457857; doi:10.2196/72812)
Supplement: Multimedia Appendix 3 [file jmir_v27i1e72812_app3.docx]

**Multimedia Appendix file 4. Coding frame based on Normalisation Process Theory**

This is an adaption of [May et al.’s (2022)](https://implementationscience.biomedcentral.com/articles/10.1186/s13012-022-01191-x) normalisation process theory coding manual, which set out NPT constructs [using a Context-Mechanism-Outcome framework](https://implementationscience.biomedcentral.com/articles/10.1186/s13012-022-01191-x/tables/1), defined them and provided examples. We have used it in our coding to guide our understanding of how NPT relates to the S&S interviews and, simultaneously, adapting the framework so that it is directly applicable to ASSIST data. Where some codes were less applicable to ASSIST data, no examples are given.

| **NPT construct** | **Sub-construct** | **Description** | **ASSIST example** |
| --- | --- | --- | --- |
| **Implementation contexts:** Contexts are patterns of social relations and structures that unfold over time and across settings. They make up the implementation environment. | **Strategic intentions** | **Description:** How do contexts shape the formulation and planning of interventions and their components? | “What was going on in terms of local government funding and we were anticipating was always going to be likely to have to be public health run, i.e. it moves out of the NHS and it’s not long before central government starts cutting it, which indeed they started doing roughly 2015, was that we were going to need to find different ways to deliver services to ensure that there was access.” |
|  | **Adaptive execution** | **Description:** How do contexts affect the ways in which users can find and enact workarounds that make an intervention and its components a workable proposition in practice? | “You just absorb [extra work] isn’t it. It’s like anything else in the NHS, you can’t really put a figure on it.” |
|  | **Negotiating capacity** | **Description:** How do contexts affect the extent that an intervention and its components can fit, or be integrated, into existing ways of working by their users? |  |
|  | **Reframing organisational logics** | **Description:** How do existing social structural and social cognitive resources shape the implementation environment? | “I think at the time there was, it was a different, to be honest we was probably more worried about our own jobs than the actual service. So there was lots of other stuff going on rather than- Because a lot of us, a lot of people including myself, their job was at risk anyway. So probably didn’t have that much interest in terms of how the service was going to be, if you like, which is a bit unfortunate but that’s the reality of that time.” |
| **Coherence:** How do people work together to understand and plan the activities that need to be accomplished to put an intervention and its components into practice? | **Differentiation** | **Description:** How did people (leaders and on the ground staff) perceive that things would be different?  What did actors to do initiate, develop and progress plans for commissioning OPSS? |  |
|  | **Communal specification** | **Description:** How did staff collectively view the rationale behind implementing OPSS?  What did they think were the reasons others had for supporting/opposing it? | “I think there was anxiety with other staff and senior clinicians, there was anxiety that if we, if xxx took off it would take away from clinic income. So I think that was like the barrier, people didn’t want to go on about it because they were scared about it. So like nothing was fed down from the top to us, because I was a Band 6 at the time, so nothing was fed down.” |
|  | **Individual specification** | **Description:** How did people individually understand what work OPSS would require of them? | **Negative Example:** “I don’t remember [the introduction of OPSS] no. Not really. It wasn’t like a big thing in the clinic. Like we didn’t get a huge filter of patients coming through there, I don’t remember it being super apparent.” |
|  | **Internalisation** | **Description:** How did people construct potential value of OPSS for their work? |  |
| **Cognitive participation:** How do people work together to create networks of participation and communities of practice around OPSS? | **Initiation** | **Description:** How did key individuals drive the implementation of OPSS?  How were stakeholders brought together in the planning, delivery or monitoring of OPSS? | “She was my consultant colleague and then she moved to [redacted]. But for her, digital health and how we would bring that in to goal and explore it was, and she was from those times and before, you know, really keen on how we explore it and do it in a systematic and robust scientific evidence that guide the delivery. But she was really keen, to bring that thinking for here.” |
|  | **Enrolment** | **Description:** How were people introduced to, and involved in, the implementation of OPSS? | “I used to always say, we're here to complement your service, we're not here to take over. It just allows you as a clinical team to see the patients that really need to be seen.”  **Negative Example:**  “It wasn’t sold to us at all, it was just like, oh you are going to start using this thing and that was it […]I think like if people had come in and like talked to us about what it was and sold it to us a bit and like explained what they do, we all would have been more engaged. But people weren’t engaged. So then what happened is people weren’t like filling in like take over care and stuff like that.” |
|  | **Legitimation** | **Description:** How do agree that OPSS is the right thing to do and should be part of STI testing delivery? | “Post-Covid, everybody prefers to test, a lot of people preferred ease of testing in the comfort of their own home. And more and more people know about xxx now so more and more people are using it. And also the clinic aren’t back up to full capacity and I don’t think they ever will be, so I think the level of people we are providing a service to will continue to increase.”  **Negative Example:**  “I do feel the way it was positioned was poor because it was potentially questioning the delivery of care that services were already doing […] to minimise and then actually reduce it to simply asymptomatic, symptomatic and tell us that thirty per cent of our work isn’t needed, I think it would affront anyone.” |
|  | **Activation** | **Description:** How do people continue to support the delivery of OPSS? |  |
| **Collective action:** How do people work together to enact OPSS? | **Interactional workability** | **Description:** How do people do the work required by OPSS? | “Only last week a patient had phoned up and gone to the chemist and they said, ‘I’m sorry we haven’t got any in’ and that was it. […] The patient said, ‘What should I do?’ She said, ‘Well phone xxx and see if they know when we’re going to get them.’ And I’m thinking a patient shouldn’t have to do that I mean I’ve already spoke to my supervisor my manager because I really felt sorry for this patient. In the end I managed to get her an appointment.” |
|  | **Relational integration** | **Description:** How does using OPSS affect the confidence that people have in each other? | “We had a lot of pressure from our Commissioners at that time, who didn't seem to understand why we weren't able to send out as many kits during that period even though it was kind of patently obvious. We didn't have the supplies and there was a national pandemic going on, but hey.” |
|  | **Skill-set workability** | **Description:** How is the work of OPSS appropriately allocated to people? | **Negative Example:** “If someone’s a pre-existing patient and they’ve got, they’ve changed their phone number, so they’ve ordered a kit and they’ve changed their phone number. They’ve put their phone number on there but the person who’s booked the kit in doesn’t update the phone number, therefore that person doesn’t get their results. And then if they’re negative, we don’t need to know about negative results, but because they don’t get their results then they call us and say, where’s my results, and it’s because someone has not done something so like simple. And then also on the flipside we get people that have got positive results then don’t get their results delaying treatment. And that person just hasn’t updated things. So and that’s nothing to do with us but we get the flack because of it.” |
|  | **Contextual integration** | **Description:** How is the work of OPSS supported by host organisations? |  |
| **Reflexive monitoring:** How do people work together to appraise OPSS? | **Systematisation** | **Description:** How do people access information about the effects of OPSS? | “Pre-pandemic we used to meet every couple of months and a core presentation around xxx to look at uptake of testing, the root of accessing, you know […] online ordering but then also the smart kits […] they looked at positivity, there was quite a bit of an issue at the beginning with false positive or reactive HIV tests so we fed back on that. So that was always a very good discussion and most of the time they responded and implemented it.” |
|  | **Communal appraisal** | **Description:** How do people collectively assess OPSS as worthwhile? | “xxx’s got something called an xxx inbox that is public. And it’s managed by our health promotion team. So anybody can go on and feedback about any xxx service. So that’s where some of those feedback came from. And then we looked at the feedback and we just changed it according to the feedback really. Because it is a service run for the people. So they need to have the vision really.” |
|  | **Individual appraisal** | **Description:** How do people individually assess OPSS as worthwhile? | “At one point people were calling and going where’s my results? So like oh there’s a delay in kits, blah blah blah blah. And then well I want my results. So then you’re like bringing them into clinic and then at some point that kit will probably get tested. So you’re just doubling the work all the time and sending the same message- So people are saying where’s my result, where’s my result? And it’s the same thing, oh there’s a delay. And we could be doing other things rather than having those same conversations all the time. So I kind of did feel that maybe they should have just stopped the home testing for a while because it wasn’t really fulfilling the purpose that it was supposed to do because you weren’t getting the kick turnaround of results.” |
|  | **Reconfiguration** | **Description:** How do people modify their work in response to their appraisal of OPSS? | During the pandemic […] we were like, ‘Look, can you take on the STI contacts because you know, we just can't see them in the clinics it is not a priority.’ So they responded, they looked at it, whether it fell into their remit, whether they feel they can provide that service safely and then if so they did it. I felt they were very good.” |
| **Implementation outcomes:** The practical effects of implementation mechanisms at work. | **Intervention performance** | **Description:** What practices have changed as the result of OPSS being operationalised, enacted, reproduced, over time and across settings? | “So with PrEP for example really we only need to see them in clinic whenever they need their kidney bloods done, which could be, for most people it’s once a year, occasionally it’s twice a year. And for their three monthly checks it could just be doing the bloods and the rest of the STI kit at home. So if they’re adherent, so obviously we review them over the phone, if they’re adherent to treatment and then we could just do it all through the phone and then can post their PrEP out of they can collect it. So it’s really good for those sorts of patients.” |
|  | **Relational restructuring** | **Description:** How has working with OPSS changed the ways people are organised and relate to each other? | “Since post-Covid with the problems we have a meeting with them every week just to make sure that they are doing what they need to do. And just to follow on to say, okay how many samples have you got left? How many kits do you have left? Do you need more kits? So it’s kind of like a triumvirate meeting.” |
|  | **Normative restructuring** | **Description:** How has working with OPSS changed the norms, rules and resources that govern action? | “we changed our appointments from being 20 minutes to being 30 minutes. And this did feed into that definitely, that we just didn’t have that let up with getting the odd asymptomatic patient” |
|  | **Sustainment (normalisation)** | **Description:** How has OPSS become incorporated in practice? |  |
