# Supplementary material for: Evaluating the Implementation of Online Postal Self-Sampling for Sexually Transmitted Infections in England: Multisite Qualitative Study
Source: J Med Internet Res. 2025 Sep 9;27:e72812. doi: 10.2196/72812 (PMC12457857; doi:10.2196/72812)
Supplement: Multimedia Appendix 4 [file jmir_v27i1e72812_app4.docx]

| **Supplementary data 2.**  **Health advisor observation sheet**  **Date** | **Site** |
| --- | --- |
| **Staff Member** | **Appointment #** |

**What happens in the appointment?**

**What do the staff member and patient discuss?**

**[When] Does OPSS come up?**

**Any other notes:**

*These scenarios and prompts were developed based on interview data with sexual health service staff, particularly those in administrative, patient-facing roles comparable to the expected participants in the Think Aloud exercises. The research team – including sexual health clinicians – reviewed the scenarios at multiple stages during their development and they were reviewed again after initial use at the first site.*

**AIMS**

- To understand how OPSS features in reception’s staff thinking about their work
- To understand how different scenarios affect how they talk about OPSS

**SCENARIOS**

**Scenario 1**

A patient walks into the clinic, approaches you at reception and tells you that they want to get tested for STIs. // [They appear to you to be an adult man.]

- What would you be thinking in this moment?
- What would you say to the patient?

The patient tells you that they have used a self-sampling kit in the past and found it difficult to collect a blood sample. // They want to get tested in a clinic.

- What would you be thinking in this moment?
- What would you say to the patient?
- Would you have had the same response before the COVID-19 pandemic?
- Would you have had the same response during the height of the COVID pandemic?

**Scenario 2**

A patient walks into the clinic, approaches you at reception and tells you that they want to get tested for STIs. They say they have never been tested before. // [They appear to you to be a teenager.]

- What would you be thinking in this moment?
- What would you say to the patient?

The patient says that they can’t wait long to be seen that day.

- What would you be thinking in this moment?
- What would you say to the patient?
- [When] would you suggest that they use OPSS?

**Scenario 3**

A patient calls the clinic and tells you they have vaginal discharge. They ask you what they should do?

- What would you be thinking in this moment?
- What would you say to the patient?

The patient’s responses do not indicate a high likelihood of an STI. They tell you they are anxious and want to be seen in the clinic.

- What would you be thinking in this moment?
- What would you say to the patient?
- Would you encourage them to use OPSS?

**Scenario 4**

A patient calls the clinic and says they have been waiting over 4 weeks for a self-sampling kit.

- What would you be thinking in this moment?
- What would you say to the patient?

The patient says they are concerned about having an STI and want to get tested as soon as possible.

- What would you be thinking in this moment?
- What would you say to the patient?
- Would you encourage them to use OPSS?
